# Document analysis of the Foundation for a Smoke-Free World’s scientific outputs and activities: a case study in contemporary tobacco industry agnogenesis

**DOI:** 10.1136/tc-2022-057667

**Published:** 2023-05-03

**Authors:** Tess Legg, Bryan Clift, Anna B Gilmore

**Affiliations:** Department for Health, University of Bath, Bath, UK

**Keywords:** Tobacco industry documents, Tobacco industry, Surveillance and monitoring

## Abstract

**Background:**

Tobacco corporation Philip Morris International launched the Foundation for a Smoke-Free World (FSFW), a purportedly independent scientific organisation, in 2017. We aimed to systematically investigate FSFW’s activities and outputs, comparing these with previous industry attempts to influence science, as identified in the recently developed typology of corporate influence on science, the Science for Profit Model (SPM).

**Design:**

We prospectively collected data on FSFW over a 4-year period, 2017–2021, and used document analysis to assess whether FSFW’s activities mirror practices tobacco and other industries have historically used to shape science in their own interests. We used the SPM as an analytical framework, working deductively to search for use of the strategies it identifies, and inductively to search for any additional strategies.

**Results:**

Marked similarities between FSFW’s practices and previous corporate attempts to influence science were observed, including: producing tobacco industry-friendly research and opinion; obscuring industry involvement in science; funding third parties which denigrate science and scientists that may threaten industry profitability; and promoting tobacco industry credibility.

**Conclusions:**

Our paper identifies FSFW as a new vehicle for agnogenesis, indicating that, over 70 years since the tobacco industry began to manipulate science, efforts to protect science from its interference remain inadequate. This, combined with growing evidence that other industries are engaging in similar practices, illustrates the urgent need to develop more robust systems to protect scientific integrity.

**WHAT IS ALREADY KNOWN ON THIS TOPIC**

- Litigation forced three tobacco industry-funded organisations to cease operating due to their role in spreading scientific misinformation.
- Philip Morris International (PMI) launched a new scientific organisation, the Foundation for a Smoke-Free World (FSFW) in 2017. Many fear FSFW plays a key scientific and public relations role for the tobacco industry.

**WHAT THIS STUDY ADDS**

- We show marked similarities between FSFW’s outputs and activities and previous corporate attempts to influence science.
- Our findings indicate that FSFW should be understood as an industry-influenced scientific lobby group promoting tobacco industry interests, akin to the historical tobacco industry-funded groups that were forcibly closed.

**HOW THIS STUDY MIGHT AFFECT RESEARCH, PRACTICE OR POLICY**

- PMI’s funding of FSFW endangers progress made in protecting science from the tobacco industry, including by rendering academic journal policies ineffective, and circumventing norms about the unacceptability of collaborating with the tobacco industry.
- The development of more robust systems to ensure science is in the public interest is urgently needed.

## Introduction

There is overwhelming evidence of the tobacco industry’s history of manipulating science—first to deny the link between cigarettes and cancer, and subsequently to deny the harms of passive smoking.^1 2^ The industry’s ability to influence science relied upon creating purportedly independent third parties to undertake key scientific roles.^3^ From the 1950s onwards, Philip Morris and others used the Tobacco Industry Research Committee (TIRC) to conduct science deflecting attention from tobacco harms^1^ and in the 1980s created the Center for Indoor Air Research (CIAR) to mislead the public about passive smoking.^2^

In the late 1990s, litigation settlements forced three tobacco industry-funded organisations based in the USA (the Tobacco Institute, TIRC and CIAR) to cease operating due to their role in spreading misinformation.^4^ A subsequent federal court order—which found the tobacco industry guilty of a ‘lengthy, unlawful conspiracy to deceive the American public’—banned US-based tobacco corporations from recreating such bodies.^5^

Since these landmark rulings, academic and public health communities have sought to better protect science from tobacco industry influence. Academics have proposed stronger firewalls between funding and research,^6^ and some scientific journals have implemented measures to manage or ban tobacco industry research.^7 8^

Despite this progress however, or perhaps because of it, in September 2017, Philip Morris International (PMI), which was not bound by the US litigation,^9^ launched a new scientific organisation, the Foundation for a Smoke-Free World (FSFW or ‘the Foundation’), pledging nearly a billion dollars in funds.^10^ With growing concern within the public health and academic communities about the nature and conduct of FSFW,^11–15^ there is a pressing need to better understand its involvement in science.

With this as our aim, we systematically assessed FSFW’s outputs and activities and compared these with strategies which diverse industries have historically used to shape science in their own interests, as identified in a recently developed evidence-based typology of corporate influence on science—the Science for Profit Model (SPM).^16^ The SPM was developed by the first and last authors, and draws on the extensive literature on corporate influence on science. It demonstrates that corporate sectors including the tobacco, pharmaceutical, chemicals, fossil fuels, alcohol and food industries have used the same collection of strategies to manufacture doubt and ignorance (or agnogenesis)^17 18^ about harms of industry products or the efficacy of policies affecting industry, promote industry-favoured solutions to public health issues and legitimise industry involvement in science.^16^ The typology outlines four macro strategies (comprising 17 meso-level strategies) through which industries have worked to influence science (see table 1). Despite other analyses providing rich accounts of the tobacco industry’s history of manipulating science,^1 18 19^ we chose to use the SPM as its comprehensive categorisation of industry strategies enables its use as an analytical framework. We address the following research questions:

- In what ways, if any, does FSFW operationalise tobacco industry influence on science?
- In what ways, if any, does PMI’s funding of FSFW jeopardise progress made to protect science from tobacco industry influence?

**Table 1:** Macro and meso strategies used by corporations to influence science as identified in the Science for Profit Model (SPM)^16^

Macro strategies*	Meso strategies
A. Influence the conduct and publication of science to skew evidence bases in industry’s favour	1. Fund and undertake ‘safe’ research
	2. Covertly undertake or prevent ‘risky’ industry research
	3. Control design and analysis of industry-funded science to ensure favourable results
	4. Shape and undermine external research
	5. Ensure favourable research is heavily represented in the evidence base
	6. Control reporting and suppress publication of unfavourable science
B. Influence the interpretation of science to undermine unfavourable science and create a distorted picture of the evidence base	7. Develop and promote criteria and concepts for critiquing science which can be used to further industry arguments
	8. Obtain and reanalyse raw data from unfavourable science
	9. Attack and misrepresent science
	10. Monitor and attack scientists and organisations
C. Influence the reach of science to create an ‘echo chamber’ for industry’s scientific messaging	11. Use legal means to protect industry evidence from being discovered or accessed
	12. Contract messengers to create scientific ‘echo chambers’
	13. Fund, produce and disseminate materials which package science in industry-favourable ways
	14. Use education, events and meetings to disseminate industry-favourable scientific messages to key stakeholders
	15. Maximise press coverage of industry-favourable scientific messages
E. Manufacture trust in industry and its scientific messaging	18. Manufacture a picture of industry credibility
	19. Conceal industry’s involvement in science, scientific messaging and influence on policy reforms that affect the use of science

*The SPM also outlines a fifth macro-level strategy which focuses on industry influence on the use of science in policy decision-making. We omit this strategy—macro strategy D, ‘Create industry-friendly policymaking environments which shape the use of science in policy decision-making in industry’s favour’ (and its meso-level strategies 16 and 17), since this was not the focus of our study.

## Method

We prospectively collected data on FSFW over a 4-year period, and used two types of document analysis to assess whether FSFW’s activities mirror previously documented industry attempts to influence science.

In September 2017, we established a system for monitoring FSFW’s outputs and activities. Beginning with FSFW’s website and relevant Google alerts (used to identify web sources), this grew to include other primary data sources, which in turn provided search terms (including names of grantee organisations and associated principal investigators) for secondary data sources (see table 2). Using these sources, we collected data related to FSFW’s work on tobacco harm reduction and smoking cessation (its agricultural diversification workstream not being the focus of this paper) until September 2021.

**Table 2:** Monitoring strategy

Type of monitoring and data sources	Types of data retrieved	Monitoring frequency
Primary sources		
1. Systematic monitoring of: FSFW website Grantee and subgrantee websites*	Strategic plans Annual reports and tax returns Blog posts Press releases Scientific reports Event information Promotional videos Lists of grantees Requests for proposals	Weekly
2. Google alerts (used to identify additional web sources) including for: ‘Foundation for a Smoke-Free World’/’FSFW’ A ‘tobacco harm reduction’ search term through which third parties were identified Key third-party organisations and individuals (grantee organisations and researchers)*	Media content (including interviews with and content written by FSFW grantees) Event information Videos of evidence to select committees	Daily
3. Personal communications (information from the tobacco control community)	Information (often emails) including: Correspondence from FSFW and its third parties to the public health community FSFW events	Ad hoc
Secondary sources		
4. Scopus Alerts for key FSFW-affiliated researchers*	Peer-reviewed publications Letters to the editor Responses to journal articles	Weekly
5. Open science (preprint) publishing platforms including BioRxiv, MedRxiv, F1000, OSF Nb. once these platforms were identified as key publication routes for FSFW-funded science, regular searches were conducted for science published by FSFW-funded researchers	FSFW-funded ‘preprint’ articles detailing: Study protocols Primary studies and reviews	Monthly
6. Altmetrics (following key FSFW-funded outputs)	Responses to FSFW research Responses to research *on* FSFW	Monthly
7. Event websites	Event funding information Speaker information PowerPoint slides of presentations	As events identified
8. LinkedIn	Profile information on: FSFW and its staff Linked organisations and individuals	Monthly

*New grantees, subgrantees and researchers were added to the monitoring lists over time.

Our analytical method was twofold. First, we drew on Forster’s approach to the analysis of company documentation,^20^ a method used in previous analyses of tobacco and food industry documents.^21–26^ This method involved understanding the meaning of individual documents through reading and rereading them over time as knowledge increases, discussing their meaning, and considering multiple documents and types of documents concurrently. The purpose of this process is to look for corroborations and discrepancies between documents to derive meaning, and the ‘back-and-forth’ between data and interpretation helps to build understanding. Documents are then recontextualised using other data sources (for instance, we compared claims made by FSFW with the wider evidence base). While Forster’s approach is primarily inductive, we conducted our analysis in a more deductive way. That is, we combined Forster’s procedural steps with a deductive approach to searching for the industry strategies identified in the SPM^16^ (using a slightly adapted version of the typology—see footnote to table 1). We also worked inductively, remaining open to identifying the use of additional strategies.

Second, through the initial stages of our analysis, it became clear that a more detailed investigation into one of the SPM’s meso strategies—‘Fund and undertake ‘safe’ research’—could bring further insights. To do this, we conducted a content analysis^27^ (rather than the iterative, comparative analysis of documents described above) of a subset of the data—peer-reviewed and preprint articles funded by FSFW. Preprint articles are outputs hosted on online open science publishing platforms (such as MedRxiv, BioRxiv and F1000). These outputs are uploaded onto the platforms by their authors, and are not subject to independent prepublication peer review. For this analysis, we used the seven types of ‘safe’ research identified in the SPM as benefiting industry as a priori categories, coding any presence of these in the dataset while also searching for new categories.

## Findings

We obtained over 700 items of data, and through our analysis found marked similarities between FSFW’s activities and outputs, and strategies previously used by corporations and their third parties to influence science. Key evidence is outlined below.

### Strategy A: influence on the conduct and publication of science

The original 2018 ‘pledge agreement’ between FSFW and PMI indicates that FSFW’s funding is conditional on its research focusing on ‘tobacco harm reduction’,^28^ rather than on broader tobacco control measures. In 2020, this document was updated. A comparison of the original and updated versions of the agreement shows the description of FSFW as ‘free from influence’^28^ from PMI was changed to ‘free from improper influence’^29^ and the following was added:

Nothing in this section… shall be interpreted to prohibit the Foundation from exchanging information or interacting with any third party, including but not limited to the pledgor… [i.e. PMI] …, or other donors, in order to advance the Foundation’s purpose.^29^

This suggests PMI is exerting, and reserves the right to exert, influence over FSFW. Collectively, FSFW-funded research outputs remain within the narrow research field dictated by this pledge agreement. Through a content analysis of FSFW-funded peer-reviewed and preprint research outputs, we found evidence of all seven of the types of ‘safe’ research (strategy 1) identified in the SPM. Such ‘safe’ research benefits industry by distracting attention from industry harms, framing industry products as ‘solutions’ and promoting interventions that minimise damage to product sales (see table 3 for illustrative examples).

**Table 3:** Funding and undertaking ‘safe’ research—content in FSFW-funded peer-reviewed and preprint articles which distracts attention from industry harms, frames industry products as part of the ‘solution’ and promotes interventions that minimise damage to product sales

Types of ‘safe’ industry research as identified in the Science for Profit Model (SPM)*	Illustrative examples from FSFW-funded research
1. Suggests causes of harm other than that of the corporate product or practice	**Detracts attention from industry harms including by:** **Pointing blame at:** **Public health:** ‘the stubbornness of smoking rates can be attributed, in part, to a neglect of adult tobacco users and the dearth of ambition among those within the public health community’^45^ **The media:** for ‘selective coverage’ on nicotine and ‘spreading misleading stories’ concluding this could impact cessation rates and public health^40^ **Omitting tobacco industry actions in explanations of why people smoke:** ‘The motivation to use tobacco involves a complex interplay between learnt and conditioned behaviours, genetics, social and environmental factors, and nicotine dependence’^31^
2. Suggests problems of corporate harm are problems ‘of the individual’	**Focuses on individuals including by:** **Asking survey questions focused on individual-level motivations to smoke, rather than external factors** (such as industry advertising, cigarette packaging, etc): ‘a majority of smokers smoked after meals (62.2%), and many also smoked every time they had coffee or tea (46.1%), or an alcoholic beverage (43.6%). Smokers were also tempted to smoke when they saw others smoking nearby (41.9%)…more than 60% of smokers and ex-smokers…had bought cigarettes when they knew the money could be spent better on household essentials like food.’^34^
3. Focuses on reducing harm from, rather than intake/use of products/practices	**Promotes ‘tobacco harm reduction’ rather than other tobacco control measures which would reduce consumption of industry products, for example:** ‘For those of us committed to tobacco harm reduction, there is no turning back—we will advocate for our patients, families, friends and fellow world citizens for their right to avail themselves of snus, heated tobacco products and e-cigarettes.’^42^
4. Suggests supposed *benefits* of industry products or practices	**Suggests potential benefits or rewards of tobacco or nicotine, for example:** Characterising the ‘benefits’ of nicotine as improving cognitive processes and mental health conditions, and emphasising rewards of tobacco smoking, for example, ‘the obvious fact so often overlooked is that smoking is rewarding and people like to do it.’^42^
5. Focuses on industry products as solutions to public health problems (rather than broader public health interventions)	**Detracts attention from broader public health interventions by promoting industry products as solutions including by focusing on:** **Tobacco and nicotine products** such as e-cigarettes and heated tobacco products (**see 3**). **Products produced by other industries** including the pharmaceutical industry (eg, smoking cessation medication)^39^ and technology industry (eg, mobile apps for smoking cessation).^106 115^
6. Suggests regulation of industry products or practices is undesirable	**Frames tobacco control policy and regulation as undesirable,^37 42 43 45 106^ labelling it as:** **Ineffective:** ‘52% of the world is ‘covered’ with respect to pack warnings, which do little to reduce smoking rates’^45^ **Regressive:** ‘regressive tobacco control policies that compound financial insecurity, such as increasing the price beyond affordable levels or fining people for smoking, and policies that criminalise use or possession, risk worsening the very conditions contributing to higher smoking rates among marginalised groups’^43^ **Having unintended consequences:** ‘tobacco harm reduction products are subject to bans in various countries…. Not only do bans preclude the adoption of harm reduction strategies but also they can foster a black market for the products. For example, Australia’s ban on nicotine e-cigarettes has given rise to a black market for nicotine liquids’^42^
7. Promotes industry as part of the solution	**Attempts to legitimise the tobacco industry:** **As a stakeholder in science:** ‘though there exists understandable leeriness about engaging with big tobacco, these companies may play a key role in funding cessation and harm reduction research’^45^ **As a stakeholder in policymaking:** ‘regarding alternative nicotine products, manufacturers need to work with policymakers to create and comply with regulatory frameworks that ensure consumer safety and quality assurance and prevent youth uptake’^39^

*We did not find additional types of ‘safe’ research beyond those identified in the SPM.

FSFW, Foundation for a Smoke-Free World.

While it is not surprising that literature reviews on newer tobacco and nicotine products often include tobacco industry-funded research (since this comprises much of the current evidence base), several FSFW-funded literature reviews rely on tobacco industry-funded literature without acknowledging its funding source, and fail to detail how literature was selected for inclusion. Such reporting omissions create the risk that literature has been cherry-picked for inclusion, potentially mirroring previous industry attempts to influence the findings and conclusions of research syntheses (strategy 3). They also have the effect of obscuring the provenance of the included works, with readers unaware that a review’s findings and conclusions are based on science including that funded by the tobacco industry. One narrative review on e-cigarettes and respiratory health^30^ emphasised potential benefits of e-cigarettes, citing literature including that funded by British American Tobacco, Philip Morris USA, Lorillard, R.J. Reynolds and Imperial Tobacco-owned Fontem Ventures. This was only evident on inspection of the cited works’ funding declarations. A preprint systematic review of the relative risks of ‘nicotine products’^31^ commissioned by FSFW^32^ failed to list the included studies (as recommended by Preferred Reporting Items for Systematic Reviews and Meta-Analyses guidelines),^33^ making it impossible to determine the extent upon which industry-funded science was relied. FSFW bases its classification of nicotine products on this preprint, making no reference to its non-peer-reviewed status.^32^

Various FSFW’s activities have helped ensure research favourable to the tobacco industry is heavily represented in the evidence base (strategy 5). FSFW and its grantees often self-publish reports on their websites or use open science (‘preprint’) publishing platforms, creating an evidence base which has not had its robustness assessed through independent peer review. On one preprint platform, F1000, where authors invite reviewers who are required to disclose conflicts of interest (COIs), FSFW invited its own grantee who gave a wholly positive review (with no COI disclosure). In contrast, the other reviewer flagged several revisions needed.^34^

Several journals which have published FSFW-funded articles had FSFW-affiliated researchers in editorial positions. Between May and July 2020, *Drugs and Alcohol Today* published a serialised special issue on the Framework Convention on Tobacco Control (FCTC), comprising nine papers all authored by FSFW grantees or staff members.^35^ Both the editor-in-chief and the guest editor had financial links to FSFW, COIs which went undeclared by the journal in relation to their editorial roles.^36^ While it is unclear whether these connections improperly influenced the publication of these articles, in February 2021, all nine articles had an expression of concern added by the publisher because of ‘credible concerns’ about editorial processes.^37–45^ In 2022, *Drugs and Alcohol Today* was renamed *Drugs, Habits and Social Policy*.^46^ The previous editor-in-chief is no longer in that role, but as of April 2023 remains a member of the editorial board.^47^ It is unclear whether the publisher's investigation is ongoing.

This was FSFW’s second known attempt to publish a special issue on this topic, the first cancelled by the *International Journal of Environmental Research and Public Health* once the managing editor understood FSFW’s tobacco industry connections.^36^ Documents concerning that special issue show that FSFW’s public relations firm, Ruder Finn, emailed the journal asking that FSFW’s president be permitted to choose contributing authors from FSFW’s grantees (University of Bath’s Tobacco Control Research Group (TCRG) personal communication, 2019). While it may be common practice for an editor of a special issue to choose its papers, a tobacco industry-funded organisation controlling the content of a special issue on the FCTC (which the tobacco industry has fought to disempower)^14 48^ is a clear conflict.

### Strategy B: influence on the interpretation of science

FSFW staff and grantees have attacked research which paints the tobacco industry in a bad light (strategy 9). In the 1990s, the industry adopted the phrase ‘junk science’ to censure science deemed unfavourable.^16^ This phrase has recently been invoked by both PMI,^49^ and by FSFW grantees, with one grantee organisation characterising concerns about e-cigarettes as ‘a fear-driven crusade’ of ‘lies and junk science’.^50^ FSFW staff and grantees have also misrepresented evidence on tobacco and nicotine products. One grantee discounted the evidence base on secondhand smoke to the New Zealand Health Select Committee when arguing against banning smoking in cars, saying ‘scientific studies have not proven that exposure to cigarette smoke in the car causes disease’.^51^ Overwhelming evidence as far back as the 1950s identifies secondhand smoke as a health risk,^52–57^ and newer evidence demonstrates that smoke-free policies lead to reductions in health harms.^58 59^ In an invited comment in the *American Journal of Public Health*,^60^ FSFW staff misrepresented evidence on the role of flavours in youth e-cigarette use, using a paper which identified flavours as the third most common reason for use^61^ to claim that flavours are not a main driver of youth e-cigarette use. Concerning the link between youth e-cigarette use and later uptake of combustible cigarettes, an article in FSFW-funded *Filter Magazine* asserted that this so-called ‘gateway’ theory had been ‘conclusively debunked’,^50^ despite the paper the article cited on this point concluding ‘the role of e-cigarettes in the future of youth smoking has yet to be definitively assessed’.^62^

FSFW and its grantees have spoken out in hostile terms against individuals and organisations that create and disseminate science unfavourable to the tobacco industry (strategy 10). They labelled authors of a report on FSFW and PMI guilty of ‘characteristic hypocrisy’ and of disseminating ‘false narratives’ about FSFW,^63^ and lamented the ‘constant (often exaggerated) bleating of public health’ about health harms of the industry’s products.^64^

### Strategy C: influence on the reach of science

FSFW and its grantees act as messengers (strategy 12), disseminating science and ‘packaging’ it in ways supporting industry interests (strategy 13) while distancing those messages from industry. FSFW has published a quarterly newsletter entitled ‘Health, Science, and Technology’,^65^ which disseminates science including that funded directly by industry,^66–68^ without making any mention of these industry links. Other ‘packaged science’ includes commentary pieces in journals (promoting industry-friendly narratives on e-cigarettes^60^ and COVID-19^69^), and evidence submissions to governments endorsing deregulatory approaches.^70 71^

FSFW and its grantees fund children’s science competitions,^72^ webinars^73^ and events (strategy 14), such as a 2020 conference where speakers^74^ presented findings from the FSFW-led special issue of journal *Drugs and Alcohol Today*,^35^ and FSFW’s PR firm, Ruder Finn, invited selected media (TCRG, personal communication, 2020). Another event with links to FSFW, the annual Global Forum on Nicotine,^75^ has provided a platform for tobacco corporations and industry-linked researchers to disseminate their science to, and build relationships with, those working independently from the industry.^76 77^

FSFW has funded media outlets which disseminate industry-friendly scientific messages (strategy 15), including *Filter Magazine* and *Vida News*, which between them have received or had approved funding of over US$1.3 million since 2018.^78–81^ Over this same period, *Filter Magazine’s* funders have also included PMI, Altria, Reynolds American, Juul Labs and FSFW grantee Knowledge Action Change.^82^ These outlets cite FSFW staff, grantees and subgrantees^83–88^; report scientific events linked to FSFW^89 90^; and disseminate both FSFW-funded research^91 92^ and critiques of science which may threaten the tobacco industry.^50 93^

An organisation with links to FSFW-funded researchers^30 94^ has also influenced what messages are *not* received by journalists. The Consumer Advocates for Smoke-Free Alternatives Association worked to prevent a journalist speaking to tobacco control researchers. In September 2019, an email read ‘in the hope that… [the journalist] …doesn’t discover the… [University of] …Bath tobacco control people on her own, I offered to do a little of the legwork for her’. (TCRG, personal communication, 2019).

### Strategy E: manufacturing trust in industry and its scientific messaging

FSFW promotes the tobacco industry’s credibility and its role in science in diverse ways (strategy 18). First, FSFW frames tobacco industry involvement in science and policy as the ‘solution’,^37 45^ and its exclusion as counterproductive. FSFW’s (now former) president condemned ‘entrenched hostility towards industry’,^95^ arguing industry-funded research is ‘robust’ and should ‘not be shunned simply on the basis of who executed or funded it’.^96^ This stands in contrast to his previous statement (before taking up this post at FSFW) that ‘academic naivete about tobacco companies’ intentions is no longer excusable’.^97^ FSFW has misleadingly likened itself^36^ to tobacco control organisations which either receive no funds from the tobacco industry^98^ or are funded by legally binding tobacco industry payments to the US government.^99 100^ Although FSFW repeatedly asserts^101–103^ that it closely adheres to criteria^6^ laid out for using tobacco industry funding for research, the authors of these criteria have specifically indicated that it does not.^104^

Conversely, despite FSFW citing transparency as one of its key tenets,^105^ its own activities (and that of its grantees) often obscure its industry links (strategy 19), thus increasing the perceived legitimacy of its science and advocacy. Several articles and commentaries lacked declarations explicitly outlining the output’s funding from FSFW when published,^38 42 106–111^ despite FSFW listing them as its publications.^112–114^ Even when a publication’s links to FSFW are made clear, FSFW’s links to PMI are often undisclosed.^34 39 40 44 115–123^

Beyond scientific publications, FSFW’s funding of one major grantee launched several subgrantee organisations positioned as experts on the science and policy of tobacco, none of whom mentioned FSFW or PMI on their websites.^124–129^ In 2020, FSFW distributed grant funds to establish ‘The Lung Trust’, ‘for the application, receipt and administration of future grant awards’,^130^ suggesting the complex network of organisations indirectly funded by PMI is likely to become ever more opaque.

## Discussion

This study—within which we took a prospective approach, collecting data over 4 years—is the first systematic and comprehensive investigation of FSFW’s outputs and activities. It is also the first paper to use the SPM as an analytical tool to investigate a contemporary industry-funded scientific organisation. Our analysis revealed that in just its first 4 years, the organisation and its affiliates have already engaged in activities which mirror all four of the SPM’s macro (and many of the meso) strategies previously used by industries to influence science. FSFW and its grantees have:

- Produced research and opinion which supports tobacco industry interests by: side-lining evidence-based tobacco control measures and endorsing interventions which ensure the sale of industry products^42 43 45 123 131^; advocating for tobacco industry involvement in science and policymaking^39 45^; and misrepresenting evidence on tobacco and nicotine products.^50 51 60^
- Published research which obscures PMI’s involvement.^34 39 40 44 106 109 115–123^
- Funded media outlets^78 80 81^ which frequently denigrate science that may jeopardise industry profitability.^50 93^
- Rallied against researchers and advocates working in tobacco control.^63 64^
- Pushed for renormalisation of the tobacco industry.^95 96^

The SPM identified that diverse industries have used these practices to achieve three proximal outcomes: (1) to create doubt about the harms of industry products, or the necessity or efficacy of policies which would affect industry; (2) to promote industry products as solutions to public health problems, and to promote industry-favoured policy responses; and (3) to legitimise the role of industry in the creation and use of science. Our analysis suggests that the launch of FSFW, and its subsequent outputs and activities, have served to help PMI, and the tobacco industry more broadly, realise these same outcomes.

Collectively, our findings indicate that FSFW should be understood as an industry-influenced scientific lobby group promoting tobacco industry interests, akin to historical tobacco industry-funded groups forcibly closed^132^ and contemporary organisations promoting the interests of the sugar,^133^ alcohol^134^ and pesticides^135^ industries. This case study adds to the body of evidence that these scientific third-party organisations play a key, and often hidden, role in operationalising industry influence on science.

FSFW is an effective vehicle for agnogenesis, not only about the evidence base on the safety and efficacy of industry products, but also about which public health solutions are optimal for society (framing consumption of industry products as essential for progress and health), and about what industry’s role should be in science and policymaking (despite evidence illustrating that industry involvement in these arenas brings negative consequences to society).^136 137^ Corporations and their third parties often conceal their agnogenic practices behind ‘superficially coherent’^138^ arguments—in this case, FSFW’s pronouncements of transparency and independence. References to agnogenesis by FSFW-funded researchers serve to redirect attention away from tobacco industry-created ignorance, with one lamenting the current ‘topsy-turvy era in which the truth is framed as a lie and lies are believed as if they are true’.^70^

### Strengths and limitations

We illustrate the breadth of FSFW’s activities and outputs, demonstrating that PMI’s influence on science goes far beyond creation of its own evidence (which has recently again seen its robustness questioned).^139 140^ We also demonstrate the relevance of the SPM to contemporary tobacco industry involvement in science—highlighting that science continues to be an important component of the industry’s political strategy, and corroborating previous investigations^16 141 142^ which concluded that science is a ‘critical resource for contemporary corporations in managing their relationships to their critics’.^142^

We make no claims about whether FSFW and those it funds are intentionally working to further the tobacco industry’s interests, but instead show how it can work to that effect. Although FSFW argues that PMI’s funding has no effect on its research,^63^ evidence shows that financial links can create an ‘implicit demand’ for researchers’ work to benefit the funder, and those in receipt of funds can respond to such pressures unintentionally and subconsciously.^143^ Further, although all researchers rely on personal interests and experiences to shape their research, financial COIs, specifically, act as a ‘megaphone, amplifying a set of interests that align with the sponsor’s’.^144^ Despite FSFW claiming a ‘confluence’ rather than ‘conflict’ of interest exists (with funder and researchers similarly striving for reduced harm from tobacco),^145^ the WHO’s FCTC asserts there is an ‘irreconcilable conflict’ between the tobacco industry’s interests and public health.^146^

Similarly, it was not the function of this paper to draw conclusions on any potential role (or otherwise) of the industry’s newer products in reducing tobacco harms. Rather, with our case study adding to growing evidence that corporate involvement in science continues to bring deleterious effects, we reiterate the standpoint^147^ that a distinction must be made between products (some of which may play a role in tackling the tobacco epidemic) and producers (who should play no role in tobacco control science and policymaking).

Where we did not find evidence of a strategy, this may be because FSFW is not engaging in such activities, or because our analysis mainly relied on publicly available documents (and was therefore unlikely to find evidence of covert activity). Such ‘gaps’ also indicate areas (including funding of medical education^148^ and links with authors of clinical practice guidelines^149^) where ongoing monitoring could be focused. Conversely, we found evidence of a relatively new^150^ scientific communication route not identified in the SPM—dissemination of industry-funded science through preprint platforms (and later citation of such without mention of its non-peer-reviewed status). This echoes historical tobacco industry activity—funding symposia in order to create scientific outputs and subsequently cite them as if peer reviewed.^2 16^

### Implications for research, policy and practice

The SPM needs to be applied to additional investigations of industry involvement in science, in order to further test and develop the model. Future research could also focus on the SPM’s strategy D (‘Create industry-friendly policymaking environments which shape the use of science in policy decision-making in industry’s favour’). While this was not the focus of the current study, we did note FSFW’s espousal of a risk-based (rather than precautionary) approach to policymaking.^73^ FSFW frames such an approach as ‘science based’^151–155^ arguing governments should ‘shift away from prohibitionist policies to more empathetic and science-based policies’.^151^ This mirrors previous tobacco industry pushes for so-called ‘science-based’ policymaking, which in the 1990s included covert attempts to inhibit policymakers’ abilities to use whole evidence bases in regulatory decision-making on corporate products.^16^ FSFW’s denigration of precautionary approaches to policymaking indicates the potential for the organisation to be used as a conduit for similar attempts.

PMI’s funding of FSFW endangers progress made in protecting science from tobacco industry influence in several significant ways. First, FSFW undermines proposed standards^6^ for using tobacco industry funding for research. By claiming to meet these standards, it disingenuously positions itself, an industry-funded scientific organisation founded with no external oversight, as the solution to industry influence on science.

Second, PMI channelling research funds through FSFW sidesteps—and thus renders ineffective—policies adopted by a growing number of academic journals which intend to prohibit publication of tobacco industry-funded science and/or mandate declaration of author COIs.^7 8 156 157^ Such policies require industry-funded researchers to be fully compliant in their disclosures (we show this was rarely the case in FSFW-funded science and research) or require journal editors to be fully informed of scientific organisations’ connections to the tobacco industry (which is virtually impossible given our finding of the growing network of grantees and subgrantees).

Further, FSFW circumvents norms about the unacceptability of collaborating with the tobacco industry, jeopardising the industry denormalisation achieved since the forced closure of the historical industry-funded scientific organisations. The *American Journal of Public Health’s* invitation to FSFW staff to comment on tobacco regulatory issues,^60^ the University of California’s approval of grant funding from FSFW^158^ and the Conrad Foundation’s acceptance of FSFW funds for its children’s science competition^159^ are unlikely to have occurred had the funding come directly from a tobacco company: equivalent relationships with PMI would not have been deemed normatively appropriate. Such decisions augment PMI’s recent direct attempts to normalise its presence in science and policy spheres.^160 161^

It is crucial that decision-makers in research, education, academia, policy and practice are aware of the role third-party organisations such as FSFW play in corporate influence on science. Beyond this, our findings indicate that over 70 years since the tobacco industry began to manipulate science, efforts to protect science from tobacco industry interference remain inadequate. The development of more robust systems to better protect scientific integrity is urgently needed.

## Data Availability

All data relevant to the study are included in the article or uploaded as supplemental information.
